# Supplementary material for: Shared genomic outliers across two divergent population clusters of a highly threatened seagrass
Source: PeerJ. 2019 Apr 29;7:e6806. doi: 10.7717/peerj.6806 (PMC6497040; doi:10.7717/peerj.6806)
Supplement: Supplemental Information 1 — Supplementary methods contain full outlier identification methods; Figure S1 Clustering analysis of the twelve sites estimated in BAPS for only neutral loci, with all twelve sites falling into one cluster; Figure S2 Ensemble model projections of probability of habitat suitability for the present-day (top) and the LGM (bottom), with surveyed estuaries represented by blue circles. Probability of suitable habitat is represented by the red to grey colour gradient; Table S1 Pairwise F ST values estimated among the 12 sampling sites (refer to Table 1 for full names of abbreviations) for the neutral dataset; Table S2 Pairwise F ST values estimated among the 12 sampling sites (refer to Table 1 for full names of abbreviations) for the complete dataset; Table S3 Candidate outliers in each site identified by PCAdapt, BayeScan, Lositan, BayeScEnv, or combinations thereof. GO terms and blast hit sequences are given where matches could be made (refer to Table 1 for full names of abbreviations). [file peerj-07-6806-s001.docx]

## Phair, N. L., Toonen, R., Knapp, I. and von der Heyden, S. Shared genomic outliers across two divergent population clusters of a highly threatened seagrass. Ecography.

## Supplementary Methods

### Outlier loci identification

The following approaches utilized the complete simulated dataset to identify outlier loci. Firstly, an F_ST_ based approach to detect putative outlier SNPs was implemented in BayeScan v2.1 (Foll & Gaggiotti 2008), which implements a Bayesian approach to directly estimate the probability that each locus is under selection using a reversible-jump Monte Carlo Markov chain (MCMC). This was carried out with a prior odds ratio of 10, 20 pilot runs, burn-in of 50,000 iterations, thinning interval of 50 and a sample size of 5,000. Secondly, the Beaumont & Nichols Fdist approach (Beaumont & Balding 2004) was implemented in Lositan (Antao et al. 2008). This was carried out using 1,000,000 iterations and an FDR of 0.05. Loci with an unusually high or low F_ST_ value, conditional on heterozygosity, are considered as potentially under selection. Thirdly, a genotype-environment correlation approach was implemented in BayeScEnv (de Villemereuil & Gaggiotti 2015), which tests for association between allele frequencies and environmental variables (Table 2). Although this method is based on the F model, it is able to consider two locus-specific effects; one due to divergent selection and another due to several processes other than local adaptation (e.g. range expansions, differences in mutation rates across loci or background selection) (de Villemereuil & Gaggiotti 2015).

Additionally, allele frequencies from the non-simulated dataset were analysed by means of a principle component analysis in the *pcadapt* package (Luu et al. 2016) in R. As this method is designed to analyses NGS data using the robust Mahalanobis distance, it is less computationally intensive and therefore faster than approaches using Bayesian statistics. Further, Luu et al. (2016) found that their method produces fewer false positive results than, for example, BayeScan. This software presented an interesting opportunity to compare the analyses implemented with the simulated and non-simulated datasets.


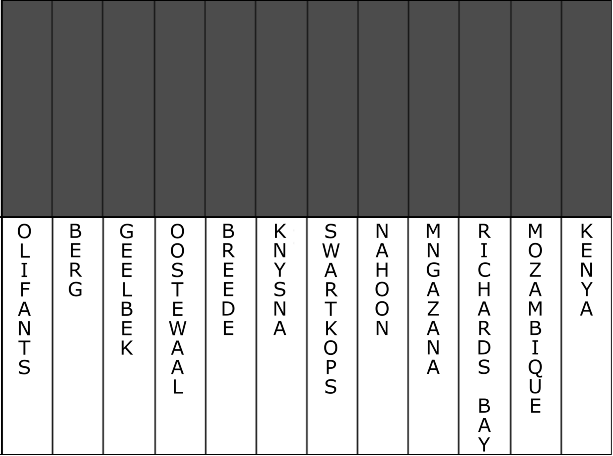
**Figure S1** Clustering analysis of the twelve sites estimated in BAPS for only neutral loci, with all twelve sites falling into one cluster.


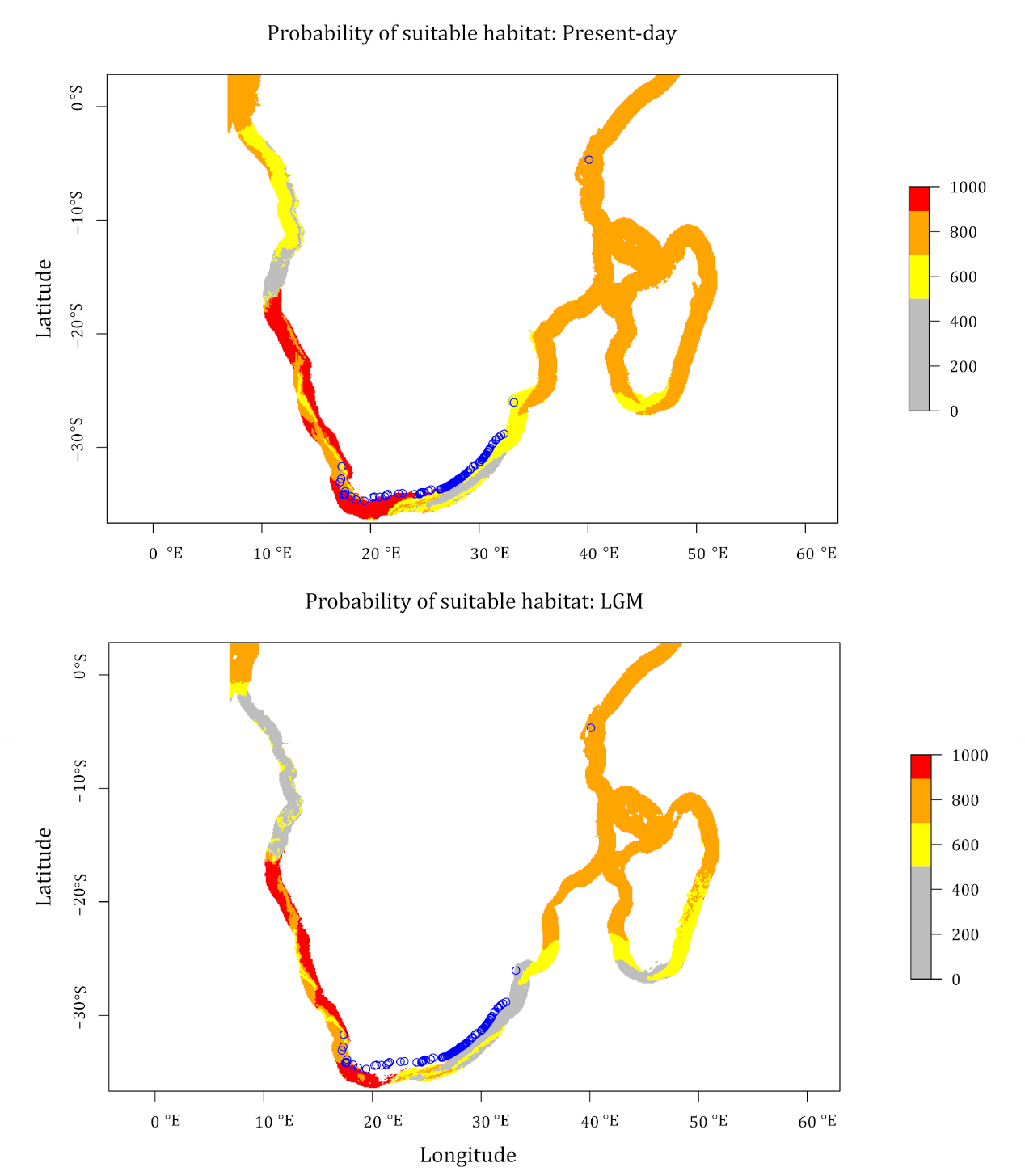


**Figure S2** Ensemble model projections of probability of habitat suitability for the present-day (top) and the LGM (bottom), with surveyed estuaries represented by blue circles. Probability of suitable habitat is represented by the red to grey colour gradient.

**Table S1** Pairwise F_ST_ values estimated among the 12 sampling sites (refer to Table 1 for full names of abbreviations) for the neutral dataset.

|  | **O** | **B** | **L1** | **L2** | **BR** | **K** | **SK** | **N** | **M** | **RB** | **MOZ** | **KEN** |
| --- | --- | --- | --- | --- | --- | --- | --- | --- | --- | --- | --- | --- |
| **O** | - |  |  |  |  |  |  |  |  |  |  |  |
| **B** | 0.017 | - |  |  |  |  |  |  |  |  |  |  |
| **L1** | 0.018 | 0.015 | - |  |  |  |  |  |  |  |  |  |
| **L2** | 0.040 | 0.043 | 0.041 | - |  |  |  |  |  |  |  |  |
| **BR** | 0.024 | 0.019 | 0.020 | 0.035 | - |  |  |  |  |  |  |  |
| **K** | 0.019 | 0.016 | 0.016 | 0.033 | 0.018 | - |  |  |  |  |  |  |
| **SK** | 0.021 | 0.017 | 0.015 | 0.042 | 0.022 | 0.016 | - |  |  |  |  |  |
| **N** | 0.047 | 0.054 | 0.050 | 0.043 | 0.050 | 0.048 | 0.053 | - |  |  |  |  |
| **M** | 0.037 | 0.037 | 0.038 | 0.040 | 0.037 | 0.035 | 0.041 | 0.046 | - |  |  |  |
| **RB** | 0.050 | 0.043 | 0.048 | 0.058 | 0.050 | 0.048 | 0.049 | 0.067 | 0.037 | - |  |  |
| **MOZ** | 0.092 | 0.095 | 0.096 | 0.087 | 0.092 | 0.095 | 0.097 | 0.095 | 0.075 | 0.073 | - |  |
| **KEN** | 0.051 | 0.044 | 0.050 | 0.060 | 0.049 | 0.049 | 0.053 | 0.068 | 0.043 | 0.042 | 0.084 | - |

**Table S2** Pairwise F_ST_ values estimated among the 12 sampling sites (refer to Table 1 for full names of abbreviations) for the complete dataset.

|  | **O** | **B** | **L1** | **L2** | **BR** | **K** | **SK** | **N** | **M** | **RB** | **MOZ** | **KEN** |
| --- | --- | --- | --- | --- | --- | --- | --- | --- | --- | --- | --- | --- |
| **O** | - |  |  |  |  |  |  |  |  |  |  |  |
| **B** | 0.023 | - |  |  |  |  |  |  |  |  |  |  |
| **L1** | 0.022 | 0.019 | - |  |  |  |  |  |  |  |  |  |
| **L2** | 0.048 | 0.053 | 0.049 | - |  |  |  |  |  |  |  |  |
| **BR** | 0.027 | 0.021 | 0.021 | 0.042 | - |  |  |  |  |  |  |  |
| **K** | 0.022 | 0.021 | 0.017 | 0.041 | 0.020 | - |  |  |  |  |  |  |
| **SK** | 0.023 | 0.022 | 0.017 | 0.049 | 0.024 | 0.019 | - |  |  |  |  |  |
| **N** | 0.061 | 0.068 | 0.059 | 0.047 | 0.061 | 0.058 | 0.061 | - |  |  |  |  |
| **M** | 0.045 | 0.043 | 0.040 | 0.043 | 0.039 | 0.038 | 0.044 | 0.053 | - |  |  |  |
| **RB** | 0.059 | 0.046 | 0.050 | 0.067 | 0.048 | 0.050 | 0.054 | 0.079 | 0.042 | - |  |  |
| **MOZ** | 0.104 | 0.102 | 0.103 | 0.093 | 0.098 | 0.103 | 0.106 | 0.104 | 0.082 | 0.081 | - |  |
| **KEN** | 0.067 | 0.054 | 0.060 | 0.071 | 0.054 | 0.060 | 0.064 | 0.084 | 0.050 | 0.046 | 0.086 | - |

Table S3 Candidate outliers in each site identified by PCAdapt, BayeScan, Lositan, BayeScEnv, or combinations thereof. GO terms and blast hit sequences are given where matches could be made (refer to Table 1 for full names of abbreviations).

| **Method** | **PCAdapt, BayeScan, Lositan, BayeScEnv** | | | **PCAdapt, BayeScan, BayeScEnv** | **BayeScan, Lositan, BayeScEnv** | **BayeScan, Lositan** | |  |  | **PCAdapt, Lositan** |  |
| --- | --- | --- | --- | --- | --- | --- | --- | --- | --- | --- | --- |
| **Outlier locus** | LFYR01001803.1_1347465 | LFYR01001803.1_1347494 | LFYR01001803.1_1347502 | LFYR01001803.1_1347472 | LFYR01001803.1_1347498 | LFYR01000838.1_24989 | LFYR01001213.1_169201 | LFYR01001714.1_282241 | LFYR01001714.1_282315 | LFYR01001803.1_1347475 | Count |
| **O** | 0,005 | 0,005 | 0 | 0 | 0 | 0,02 | 0,02 | 0,017 | 0,02 | 0,005 | 7 |
| **B** | 0 | 0 | 0 | 0,004 | 0,004 | 0,033 | 0,041 | 0,03 | 0,023 | 0 | 6 |
| **L1** | 0,014 | 0,032 | 0,032 | 0,041 | 0,032 | 0,04 | 0,024 | 0,052 | 0,045 | 0,027 | 10 |
| **L2** | 0,375 | 0,359 | 0,368 | 0,35 | 0,359 | 0,104 | 0,024 | 0,054 | 0,041 | 0,45 | 10 |
| **BR** | 0,06 | 0,072 | 0,073 | 0,06 | 0,073 | 0,064 | 0,054 | 0,074 | 0 | 0,084 | 9 |
| **K** | 0,007 | 0,015 | 0,015 | 0,015 | 0,015 | 0,063 | 0 | 0,038 | 0,048 | 0 | 8 |
| **SK** | 0,013 | 0,025 | 0,025 | 0,025 | 0,025 | 0,04 | 0,028 | 0,034 | 0,06 | 0,025 | 10 |
| **N** | 0,5 | 0,361 | 0,366 | 0,37 | 0,493 | 0,066 | 0,047 | 0,022 | 0,072 | 0,493 | 10 |
| **M** | 0,1 | 0,35 | 0,438 | 0,449 | 0,359 | 0,019 | 0,029 | 0,039 | 0,039 | 0,102 | 10 |
| **RB** | 0,057 | 0,118 | 0,109 | 0,138 | 0,123 | 0,061 | 0,034 | 0,006 | 0,021 | 0,064 | 10 |
| **MOZ** | 0,118 | 0,429 | 0,444 | 0,443 | 0,429 | 0 | 0,067 | 0 | 0,048 | 0,42 | 8 |
| **KEN** | 0 | 0,394 | 0,375 | 0,438 | 0,394 | 0,079 | 0,026 | 0,033 | 0,03 | 0,02 | 9 |
|  |  |  |  |  |  |  |  |  |  |  |  |
| **Count** | 10 | 11 | 10 | 11 | 11 | 11 | 11 | 11 | 11 | 10 |  |
|  |  |  |  |  |  |  |  |  |  |  |  |
| **GO terms** | Integral component of membrane; membrane |  |  |  |  | Integral component of membrane; membrane |  | Integral component of membrane; membrane | Integral component of membrane; membrane |  |  |
| **BLAST hit sequence** | hypothetical protein CQW23_33451 [Capsicum baccatum] gi\|1270974041\|gb\|PHT26942.1\| |  |  |  |  | hypothetical protein ZOSMA_212G00120 [Zostera marina] gi\|901815149\|gb\|KMZ69491.1\|  hypothetical protein ZOSMA_71G00010 [Zostera marina] gi\|901796154\|gb\|KMZ58935.1\|  hypothetical protein ZOSMA_27G01320 [Zostera marina] gi\|901811680\|gb\|KMZ67078.1\| | hypothetical protein ZOSMA_71G00010 [Zostera marina] gi\|901796154\|gb\|KMZ58935.1\| | Predicted: uncharacterized protein LOC108221298 [Daucus carota subsp. sativus] gi\|1040882023\|ref\|XP_017250676.1\|  hypothetical protein ZOSMA_212G00120 [Zostera marina] gi\|901815149\|gb\|KMZ69491.1\| | hypothetical protein ZOSMA_212G00120 [Zostera marina] gi\|901815149\|gb\|KMZ69491.1\| |  |  |
